# Supplementary material for: Reconciling Mining with the Conservation of Cave Biodiversity: A Quantitative Baseline to Help Establish Conservation Priorities
Source: PLoS One. 2016 Dec 20;11(12):e0168348. doi: 10.1371/journal.pone.0168348 (PMC5173368; doi:10.1371/journal.pone.0168348)
Supplement: S1 Dataset — (ZIP) [file pone.0168348.s002.zip › Taxa/Serra Norte/SN_2013/N5S/Lista N5S-33.pdf]

CAVIDADE N5S-0033

Classe	Ordem	Fam/Outros	Gên/Outros	Espécie	S-U	U-S	Zon.
Arachnida	Acari	(Parasitengonina)		sp.2	X		P
Arachnida	Acari	(Parasitengonina)		sp.5		X	P
Arachnida	Acari	(Parasitengonina)		sp.6		X	P
Arachnida	Acari	Acaridae		sp.1		X	P
Arachnida	Acari	Argasidae	*Ornithodoros*	sp.1	X	X	P
Arachnida	Acari	Ixodidae	*Amblyomma*	sp.1	X		P
Arachnida	Amblypygi	Phrynidae	*Heterophrynus*	*longicornis*		1	P
Arachnida	Araneae	Ctenidae		jovem	1		P
Arachnida	Araneae	Ochyroceratidae		jovem	X	X	P
Arachnida	Araneae	Oonopidae	aff. *Matiotia*	sp.1		X	E
Arachnida	Araneae	Pholcidae	*Leptopholcus*	sp.1	X		P
Arachnida	Araneae	Pholcidae	*Mesabolivar*	*eberhard*		X	P
Arachnida	Araneae	Theridiidae	*Achaearanea*	sp.1	X		P
Arachnida	Araneae	Theridiidae		sp.5		X	E
Arachnida	Araneae	Theridiosomatidae	*Plato*	sp.1	X	X	P
Arachnida	Opiliones	Sclerosomatidae	*Prionostema*	sp.1		X	P
Arachnida	Palpigradi	Eukoeneniidae	*Allokoenenia*	sp.1	X		P
Arachnida	Pseudoscorpiones	Chthoniidae	*Pseudochthonius*	sp.1	X		P
Diplopoda	Polydesmida	Chelodesmidae		sp.2		1	P
Diplopoda	Polydesmida	Paradoxosomatidae		sp.1		1	P
Diplopoda	Spirostreptida	Pseudonannolenidae	*Pseudonannolene*	sp.1		1	P
Entognatha	Collembola	Paronellidae		sp.1		X	P
Entognatha	Diplura	Campodeidae		sp.1		X	P
Insecta	Blattodea	Blaberidae		jovem		2	P
Insecta	Coleoptera	Curculionidae	Scolytinae	sp.2		X	P
Insecta	Coleoptera	Tenebrionidae		jovem	X		P
Insecta	Diptera	Cecidomyiidae	Cecidomyiinae	sp.	X	X	E/P
Insecta	Diptera	Ceratopogonidae		jovem	X		P
Insecta	Diptera	Corethrellidae	*Lutzomiops*	sp.	X		P
Insecta	Diptera	Culicidae	*Culex*	sp.	X		P
Insecta	Diptera	Psychodidae	*Evandromyia*	*saulensis*		X	P
Insecta	Diptera	Psychodidae	*Sciopemyia*	*sordellii*	X	X	E/P
Insecta	Diptera	Tipulidae	Tipulinae	sp.	X		P
Insecta	Heteroptera	Reduviidae	Reduviinae	jovem	2	1	P
Insecta	Heteroptera	Schizopteridae	Schizopterinae	jovem	X		P
Insecta	Homoptera	Cixiidae		jovem		X	P
Insecta	Hymenoptera	Diapriidae		sp.2		X	P
Insecta	Hymenoptera	Formicidae	*Camponotus*	sp.1	X		P
Insecta	Hymenoptera	Formicidae	*Pheidole*	sp.2	X	X	E/P
Insecta	Hymenoptera	Formicidae	*Wasmannia*	sp.1		X	P
Insecta	Isoptera	Termitidae	*Nasutitermes*	sp.	X	X	E/P
Insecta	Lepidoptera	Gelechioidea		sp.4	X		P
Insecta	Lepidoptera	Noctuoidea		sp.2		X	P
Insecta	Lepidoptera	Noctuoidea		sp.12		2	E
Insecta	Lepidoptera	Pyraloidea		sp.		X	P
Insecta	Orthoptera	Mogoplistidae		sp.		1	E
Insecta	Orthoptera	Phalangopsidae	*Paraclodes*	sp.	1		P
Insecta	Orthoptera	Phalangopsidae	*Phalangopsis*	sp.		17	P
Insecta	Thysanoptera	Thripidae		sp.1		X	P

Insecta	Thysanura	Nicoletiidae		sp.1		X	P
Gastropoda	Pulmonata	Systrophiidae	*Happia*	sp.		X	P
Malacostraca	Isopoda	Armadillidae		sp.1	X	X	P
Mammalia	Chiroptera	Emballonuridae	*Peropteryx*	sp.	2	2	P
